# Role of Magnetic Resonance Imaging in evaluating T2-weighted hyperintensities in spinal cord in a tertiary care hospital in central India

**DOI:** 10.12688/f1000research.144117.2

**Published:** 2024-06-21

**Authors:** Rishabh Dhabalia, Shivali Kashikar

**Affiliations:** 1 Radio-diagnosis, Datta Meghe Institute of Higher Education & Research, Wardha, 442001, India

**Keywords:** Spinal Cord, T2-Weighted Hyperintensities, MRI Evaluation, Clinical Features, Pathological Correlation, Systematic Assessment

## Abstract

**Background:**

Hyperintensities on T2-weighted images in the spinal cord are a complex and diagnostically challenging entity that can present with diverse clinical features. This study protocol outlines a comprehensive investigation to understand the causes, clinical and imaging characteristics, and correlation with pathological findings of hyperintensities on T2-weighted images in the spinal cord. By establishing a systematic assessment approach, this study seeks to provide valuable insights into these abnormalities’ diagnostic and prognostic implications.

**Methods:**

The study will be conducted as a prospective observational design. Patients with clinically diagnosed or suspected spinal cord lesion presenting with intramedullary T2-weighted hyperintensity and referred for MRI evaluation will be included. Data collection will encompass patient demographics, clinical features, and extensive imaging parameters. Pathological data, when available, will be correlated with imaging findings. Various statistical methods will be employed to analyse the data, including frequency analysis, comparative tests, logistic regression, and survival analysis.

**Expected Results:**

The study anticipates elucidating the spectrum of etiologies underlying hyperintensities on T2-weighted images in the spinal cord and their clinical and imaging profiles. The systematic approach will offer a structured diagnostic method, while correlations with pathological data will provide an enhanced understanding of these conditions. The results are expected to provide clinicians with valuable insights into diagnosing, treating, and prognosticating patients with spinal cord hyperintensities on T2-weighted images.

## Introduction

T2-weighted hyperintensities within the spinal cord represent a diverse and challenging diagnostic entity, the understanding of which is critical for improving patient care and outcomes. ^1^ These abnormalities manifest with various clinical symptoms, making their etiological diagnosis a complex and vital task. Magnetic resonance imaging (MRI) is the cornerstone for evaluating spinal cord pathologies, offering a window into the intricate details of the spinal cord’s internal structure and any potential anomalies. ^2^ In this context, the current study protocol is designed to investigate T2-weighted hyperintensities in the spinal cord comprehensively. It focuses on identifying their causes, analysing clinical and imaging features, developing a systematic diagnostic approach, and correlating with pathological data when available.

Spinal cord pathologies can lead to many clinical presentations, from sensory and motor deficits to more complex neurological signs, impacting affected individuals’ quality of life and prognosis. The ability to accurately identify and diagnose the underlying causes of T2-weighted hyperintensities in the spinal cord is crucial for timely intervention and treatment planning. ^3^ Moreover, understanding the relationships between clinical features, imaging characteristics, and pathological findings can provide a holistic perspective on these conditions, facilitating more precise diagnosis and improved patient care. ^4^

The study aims to provide valuable insights into the spectrum of etiologies of T2-weighted hyperintensities within the spinal cord and their correlation with clinical and imaging features. Developing a systematic, algorithmic approach for their assessment will enhance diagnostic accuracy and offer a structured framework for clinicians. Furthermore, when pathological data is available, this study will bridge the gap between imaging and histopathological findings, potentially shedding light on the mechanistic aspects of these abnormalities.

Ultimately, the findings from this investigation are expected to contribute to a better understanding of T2-weighted hyperintensities in the spinal cord, facilitating early diagnosis, treatment, and improved clinical-neurological outcomes for affected patients. By addressing the complex diagnostic challenges associated with these abnormalities, this study protocol seeks to advance our knowledge and clinical management of spinal cord pathologies, further underscoring the significance of MRI evaluation in this context.

### Aim

This study aims to investigate the prevalence and impact of T2-weighted hyperintensities in the spinal cord, determine their etiological factors, establish a systematic assessment approach, and correlate clinical and imaging features with neurological outcomes.

### Objectives

1. To determine the causes of T2-weighted hyperintensities in the spinal cord.
2. To analyse the clinical and imaging characteristics associated with different etiologies of T2-weighted hyperintensities in the spinal cord.
3. Establishing a systematic, algorithmic approach for assessing intramedullary T2-weighted hyperintensities, considering the topography and extension of the abnormalities.
4. To establish a diagnostic approach considering the topography and extension of the spinal cord abnormalities.
5. To assess the morphological aspects of spinal cord hyperintensities and correlate findings with pathological data where available.

## Methods

### Study design

Prospective Observational Study. Patients with clinically diagnosed or suspected spinal cord lesion, presenting with intramedullary T2-weighted hyperintensities, will undergo an MRI evaluation of the spinal cord. Data will be collected in setup 2023-2024 period, and statistical analyses will be performed to achieve the study objectives.

### Study population

Patients with clinically diagnosed or suspected spinal cord injury, irrespective of age or gender, were referred to the Department of Radiodiagnosis, AVBRH, Sawangi, and presenting with intramedullary T2-weighted hyperintensity in the spinal cord.

### Place of study

Department of Radiodiagnosis, AVBRH (Datta Meghe Institute of Higher Education & Research), Sawangi (Wardha).

### Inclusion criteria

1. Clinically diagnosed or suspected patients with spinal cord lesion are referred to the Department of Radiodiagnosis for MRI evaluation of the spinal cord.
2. Patients with intramedullary T2-weighted hyperintensity.
3. Patients of all age groups and both genders.
4. Patients willing to undergo investigations, participate in the study, and agree to followup if required.

### Exclusion criteria

1. Patients with cardiac pacemakers, metallic implant insertion, or metallic foreign bodies in situ.
2. Patients with a history of claustrophobia.
3. Patients who refuse to provide informed consent.
4. Non-cooperative patients.

### Bias

1. **Selection bias:** There may be a potential bias in selecting study participants. If patients are not randomly selected and instead are chosen based on the severity of their condition or other non-random criteria, this can introduce selection bias. For example, patients with more severe symptoms may be more likely to undergo MRI evaluation, potentially leading to overrepresenting specific etiologies of T2-weighted hyperintensities.
***Overcoming selection bias:***
  • **Random sampling:** To reduce selection bias, employ random sampling methods to ensure that all eligible patients have an equal chance of being included in the study.
  • **Clear inclusion criteria:** Clearly define the inclusion criteria to ensure consistency in patient selection.
2. **Observer bias:** This bias can occur during the interpretation of MRI scans. If the radiologists or clinicians interpreting the MRI scans are aware of the patient’s clinical history or if they have preconceived notions about the potential cause of T2-weighted hyperintensities, this may influence their assessments.
***Overcoming observer bias:***
  • **Blinding:** Implement blinding procedures where the radiologists or clinicians interpreting the MRI scans are unaware of the patient’s clinical history and etiological factors.
  • **Inter-rater agreement:** Ensure that multiple radiologists or clinicians independently assess the MRI scans and measure inter-rater agreement to minimise bias.
3. **Information bias:** Information bias may arise if there are inaccuracies in data collection, documentation, or patient reporting. Only accurate or complete data can lead to errors in the study’s findings.
***Overcoming information bias:***
  • **Standardized data collection:** Use standardised data collection forms and procedures to ensure consistent and accurate data collection.
  • **Patient education:** Educate patients about providing accurate and complete information, including medical history and symptoms.

### Outcome

*The primary outcome of the study*:

1. **Identification of causes:** Determining the various underlying causes of T2-weighted hyperintensities in the spinal cord.

*Secondary outcomes*:

2. **Clinical and imaging features:** Analyzing the clinical and imaging characteristics associated with different causes of hyperintensities.
3. **Diagnostic approach:** Establishing a systematic, algorithmic approach for assessing intramedullary T2-weighted hyperintensities.
4. **Pathological correlation:** Correlating imaging findings with pathological data, whenever possible, to enhance diagnostic accuracy.
5. **Neurological outcomes:** Assessing the correlation between spinal cord hyperintensities and clinical-neurological outcomes, potentially aiding prognosis and treatment decisions.

### Enrollment

1. **Identification of eligible patients:** The enrollment process will begin with identifying patients who meet the inclusion criteria for the study. This includes individuals with clinically diagnosed or suspected spinal cord lesion referred to the Department of Radiodiagnosis, AVBRH, Sawangi, and present with intramedullary T2-weighted hyperintensity in the spinal cord.
2. **Patient education and informed consent:** Patients identified as potentially eligible for the study will be approached by the study investigators or healthcare providers responsible for their care. They will be informed about the study’s purpose, procedures, potential risks, and benefits. Patients will be provided with both verbal and written information about the study. They will have the opportunity to ask questions and seek clarification.
3. **Obtaining informed consent:** Patients willing to participate in the study will be asked to provide written informed consent. The informed consent process will be conducted in a private and confidential setting to ensure that patients fully understand the study and voluntarily agree to participate. Those who decline to participate or do not meet the inclusion criteria will be excluded.
4. **Proforma completion:** The healthcare providers or study investigators will fill out a proforma after obtaining informed consent. This proforma will collect relevant patient information, including demographic data, clinical history, and other essential details necessary for the study.
5. **MRI evaluation:** After enrollment, eligible patients will undergo an MRI evaluation of the spinal cord. The imaging will be performed per the standard protocols and procedures established by the Department of Radiodiagnosis.
6. **Regular monitoring:** Throughout the enrollment process, there will be ongoing monitoring to ensure that all eligible patients are approached, informed, and allowed to participate. Any challenges or issues related to enrollment will be documented and addressed promptly.
7. **Data management:** All collected data, including patient information and MRI findings, will be managed securely and confidentially, with access limited to authorised study personnel only.

### Data collection process

The data collection process for this study is a critical component that ensures the systematic and accurate gathering of information about the objectives. It involves several stages designed to maintain the highest data quality standards and ethical considerations.

1. **Patient information and informed consent:** Upon enrollment, patients who have provided informed consent will have their demographic information recorded. This includes age, gender, medical history, and any pertinent clinical details related to their spinal cord injury. The informed consent process is confidential to ensure that participants understand the study’s purpose and voluntarily agree to participate.
2. **Imaging data acquisition:** The core of this study relies on magnetic resonance imaging (MRI) to assess T2-weighted hyperintensities in the spinal cord. Imaging data will be collected using state-of-the-art MRI equipment following established protocols. This imaging process will generate detailed images of the spinal cord and surrounding structures.
3. **Clinical and imaging features:** Clinical features will be documented, including presenting symptoms, duration of symptoms, and any neurological deficits. Imaging features, such as the location, size, and characteristics of T2-weighted hyperintensities, will be recorded by experienced radiologists. The collected clinical and imaging data will provide valuable insights into the presentation and characteristics of spinal cord hyperintensities.
4. **Pathological data and correlation:** Whenever possible, pathological data will be collected through procedures like biopsies or surgeries. This data will be correlated with the imaging findings, allowing for a comprehensive understanding of the underlying causes of T2-weighted hyperintensities.
5. **Proforma completion:** Healthcare providers or study investigators will fill out a standardised proforma. This proforma will collect detailed information, ensuring the systematic and uniform documentation of all relevant patient data. It acts as a bridge between clinical and imaging information, aiding in the correlation process.
6. **Data entry and management:** Trained personnel will enter All collected data into a secure and confidential database system. Strict data entry standards will be upheld to minimise errors and ensure data integrity. Access to the data will be restricted to authorised study personnel to maintain privacy and confidentiality.
7. **Quality control and validation:** Data quality will be monitored through regular validation processes to identify any inconsistencies or inaccuracies. These will be promptly addressed to maintain the accuracy and reliability of the dataset.
8. **Ethical considerations:** Ethical guidelines and patient confidentiality will be strictly adhered to throughout the data collection process. All patient data will be anonymised to protect privacy, and the study will comply with relevant data protection and patient confidentiality regulations.

### Imaging protocol

MRI Spine protocol includes various standard & additional MRI sequences for routine assessment. The details of the protocol will vary based on the type of MRI scanner, the particular hardware and software used, the preferences of the radiologist and potentially the referring physician, patient-specific factors such as implants, the specific clinical indications, and any time constraints. Patients meeting the study criteria will undergo an MRI examination while in a supine position, utilizing either a spine coil or a table-embedded coil array. MRI acquisition is performed using a standard pulse sequence protocol.

Sequences will be manually designed, involving the setup of field of view (FOV) positioning and angulation, guided by an initial survey scan. The scan will span the entire spine from cranial to caudal direction, covering the complete diameter of the vertebrae, neural foramina, facet joints (FJs) & paraspinal muscles in transverse direction.

### Technical parameters

*Imaging configuration*

• Planar spatial resolution: ≤0.8 × 0.8 mm
• field of view (FOV): axial : 100-160 (cervical spine), 150-250 (dorsolumbar spine); sagittal/coronal: 200-240 (cervical spine), 300-400 (dorsolumbar spine)
• slice thickness: ≤ 4 mm

*Imaging planning*

• Images in sagittal plane:
  ○ angulation: along the spinal axis and the spinous processes
  ○ slice thickness: ≤4 mm
• Images in Axial plane:
  ○ angulation: perpendicular to the spine
  ○ ensure slices intersect perpendicularly with nucleus pulposus
  ○ slice thickness: ≤4 mm
• Images in Coronal plane:
  ○ angulation: parallel to the spinal axis and transverse processes
  ○ slice thickness: ≤4 mm

### Sequences

*Standard sequences*

• T1-weighted:
  ○ purpose: evaluation of bone & soft-tissue
  ○ technique: T1-FSE (fast spin echo)
  ○ planes: axial, coronal & sagittal
• T1 with contrast:
  ○ purpose: to evaluate inflammatory conditions or potential tumors
  ○ technique: T1-FSE, T1 Dixon
  ○ planes: axial, coronal & sagittal
• Contrast injection of Gadolinium-DTPA (0.1-0.2 mg/kg body weight) will be given intravenously.
• T1WI: - TR 400–550 m/s, TE 15 m/s. short repetition time and echo time
• T2-weighted:
  ○ purpose: assessment of bones, soft tissue, tendons & ligaments
  ○ technique: T2-FSE, T2 Dixon
  ○ planes: axial, coronal & sagittal
• T2-weighted-FS (fat-saturated):
  ○ purpose: assessment of bones, fractures, soft tissue & inflammatory changes, fractures
  ○ technique: T2-FS-FSE, T2 Dixon/STIR, T2 GRE (gradient echo)
  ○ planes: axial & sagittal
• T2WI: -TR 1000 m/s, TE 110 m/s. Long TR and TE. Matrix size 256 × 256,

*Additional sequences*

• chemical shift imaging
  ○ purpose: assessment of bone tumors and vertebral lesions
  ○ technique: T1 Dixon, T1 GRE
  ○ planes: sagittal
• diffusion-weighted imaging
  ○ purpose: evaluation of spinal cord ischemia, differentiation spondylodiscitis vs degenerative changes
  ○ technique: DWI/DTI
  ○ planes: sagittal
  ○ Diffusion – sensitizing gradients will be applied with a diffusion sensitivity of b = 0 , 500, 1000 s/mm ^2^
  ○ ADC values will be measured from the core and periphery of the lesion, as well as from the surrounding edema.
• MRS protocol
• Voxel size and positioning
• All cases were evaluated by multi-voxel spectroscopic technique (MVS). The voxel of size 1.6 × 1.6 × 1.6 cm ^3^ will be placed within those suspicious lesions observed in post-contrast T1-weighted imaging. To establish a reference spectrum, the voxel will be expanded to encompass the contralateral normal brain tissue

*Pulse sequence*

○ Point resolved spectroscopy will be used.
○ Parameters: TR/TE 1000/144 and 1500/35
○ Short echo time: to illustrate aspartate, Amino Acid, succinate, lipid-lactate peak & acetate peaks
○ Intermediate echo time: to illustrate Choline, Creatine, N-AcetlyAspartate and alanine peaks.

### Sample size

The formula used for sample size calculation is as follows:

$$\text{Sample size}\;\left(n\right)=\left[{\text{DEFF}}^{*}\;N^{*}\;p\left(1-p\right)\right]/\left[d^{2}/\left(Z^{2}{\left(1-\alpha /2\right)}^{*}\;\left(N-1\right)+p\left(1-p\right)\right)\right]$$

In this case, the sample size calculation was based on the following parameters:

• Population size (N): 1,000,000 (estimated)
• Hypothesized % frequency of the outcome (p): 17.5% ± 10
• Confidence limits as % of 100 (absolute ± %) (d): 10%
• Design effect (DEFF): 1
• Confidence level (1-α/2): 99%

Given these parameters, the sample size was calculated to be 96. This sample size was determined to provide a 99% confidence level with a 10% margin of error while accounting for the estimated prevalence of the outcome in the population.

### Statistical methods

For this study on T2-weighted hyperintensities in the spinal cord, a comprehensive range of statistical methods will be employed to address the research objectives. The analysis will commence with descriptive statistics to provide an overview of the data’s central tendencies and distribution. Frequent analysis will be conducted to determine the prevalence of various causes of hyperintensities. Comparative analyses will involve chi-square tests, t-tests, and ANOVA to explore associations between clinical and imaging features and different causes of hyperintensities. Logistic regression will be applied to identify predictors and risk factors, while multinomial logistic regression will help assess factors associated with multiple categories of outcomes. Additionally, linear regression will be used to explore relationships between continuous outcomes and independent variables. Cox regression will address time-to-event data, such as clinical progression. Correlation analysis, survival analysis, and factor or cluster analysis will further assist in unveiling patterns and associations within the dataset. Comprehensive data visualisation will support the interpretation of findings. Subgroup and sensitivity analyses will also be conducted to explore variations across demographic or clinical subgroups and test the robustness of the results. Specialised statistical software will facilitate data analysis, and collaboration with a statistician will ensure these statistical techniques’ rigorous and appropriate application by using R Studio Version 4.3.1.

### Ethical considerations

The Institutional Ethics Committee of Datta Meghe Institute of Higher Education and Research (DU) has granted its approval to the study protocol (Reference number: DMIHER (DU)/IEC/2023/545. Date:03-02-2023). Before commencing the study, we will obtain written informed consent from all participants, providing them with a comprehensive explanation of the study’s objectives.

### Dissemination

After the completion of the study, we will publish it in an indexed journal or conference.

### Study status

The study has yet to start after the publication of the protocol; we will start recruitment in the study.

## Discussion

As outlined in this study protocol, the evaluation of T2-weighted hyperintensities in the spinal cord holds significant clinical relevance. These hyperintensities present a diagnostic challenge due to their varied etiologies and clinical manifestations, necessitating a structured approach for assessment and correlation with pathological findings.

The systematic investigation of these intramedullary hyperintensities is aligned with the growing recognition of magnetic resonance imaging (MRI) as the gold standard for spinal cord evaluation. MRI offers high sensitivity, excellent contrast resolution, and the ability to visualise soft tissue structures in multiple planes, making it indispensable in assessing spinal cord disorders. ^5^ Our study’s emphasis on T2-weighted hyperintensities extends its significance, as these abnormalities may harbour crucial clinical information.

The clinical and imaging features of T2-weighted hyperintensities in the spinal cord are central to understanding the impact on patients. The proposed study aims to provide insights into the characteristics associated with different etiologies of these hyperintensities. The ability to correlate imaging features with clinical symptoms can aid in early diagnosis and guide treatment decisions. Previous research has emphasised the diagnostic potential of MRI in identifying various spinal cord pathologies, including traumatic injuries, disc herniation, inflammatory, and demyelinating conditions. The main imaging patterns of demyelinating myelopathies (multiple sclerosis, neuromyelitis Optica spectrum disorder, acute disseminated encephalomyelitis, and myelin oligodendrocyte glycoprotein antibody-related disorder) and inflammatory myelopathies (systemic lupus erythematosus-myelitis, sarcoidosis-myelitis, Sjogren-myelitis, and Bechet's-myelitis) have MRI features that can aid to obtain the right diagnosis. ^6^^–^^8^ Our study seeks to contribute to this body of knowledge by examining the specific characteristics associated with T2-weighted hyperintensities.

Moreover, developing a systematic, algorithmic approach for their assessment aligns with the call for standardised diagnostic protocols. A structured approach can improve diagnostic accuracy and consistency, ensuring patients receive appropriate care promptly. The utility of systematic approaches has been recognised in diagnosing spinal cord injuries and associated neurological deficits. ^9^ In this context, our study aims to provide a framework that can be integrated into clinical practice, facilitating more precise and timely diagnoses.

The correlation of imaging findings with pathological data, whenever available, further enhances the study’s significance. This linkage between radiological and histopathological data may provide valuable insights into the underlying mechanisms of these hyperintensities.

## Data Availability

No data are associated with this article.
