# Supplementary material for: Role of Magnetic Resonance Imaging in evaluating T2-weighted hyperintensities in spinal cord in a tertiary care hospital in central India
Source: F1000Res. 2024 Jul 23;13:502. Originally published 2024 May 17. [Version 3] doi: 10.12688/f1000research.144117.3 (PMC11844792; doi:10.12688/f1000research.144117.3)
Supplement: Peer review history

# Reviewer report: Reviewer response for version 3

**DOI:** 10.5256/f1000research.169442.r364472

**Published:** 2025-02-19

**Authors:** Dario Pfyffer

**Affiliations:** 1 Department of Anesthesiology, Perioperative and Pain Medicine, Stanford University School of Medicine, Palo Alto, USA

This comprehensive study protocol nicely describes the aim to characterize magnetic resonance imaging-derived T2-weighted intramedullary hyperintensities and their association with injury etiologies and clinical characteristics. The goal is to establish a standardized diagnostic approach for assessing these hyperintensities and their morphological aspects, their relationship to pathological data, and their neurological/clinical prognostic value.

 General: please be consistent with capital letters (e.g., manuscript title, first sentence of the methods ‘Prospective observational study’); I do not recommend mixing lower and upper cases.

 Please also be consistent with regard to abbreviations. Do not introduce them multiple times (e.g., MRI).

 Title: shorten to something like ‘Using T2-weighted magnetic resonance imaging to evaluate spinal cord hyperintensities in a central India tertiary care hospital.’

 Abstract, methods: please rephrase ‘The study will be conducted as a prospective observational design.’ E.g., …conducted using a….

 Abstract, methods: ‘…presenting with intramedullary T2-weighted hyperintensity and referred for MRI evaluation…’ sounds confusing. Presence of T2-weighted hyperintensities can only be confirmed after the MRI. Would thus rephrase this sentence. Or has T2-weighted hyperintensity already been confirmed at an earlier MRI? Similarly for the study population section and inclusion criteria section (second point) under methods. This should be made clear and adapted throughout the manuscript.

 I would love to see some examples of clinical and imaging features, pathological data, and neurological outcomes planned to assess. Please elaborate a bit if possible.

 For the MRI spine protocols, it would be great to get some more details, such as magnetic field strength and coils used, even if the hardware will be varying (different models).

 Please include the following two references in the introduction (second section, along with reference 3): [ ref 1 and 2]

Is the study design appropriate for the research question?

Yes

Is the rationale for, and objectives of, the study clearly described?

Yes

Are sufficient details of the methods provided to allow replication by others?

Partly

Are the datasets clearly presented in a useable and accessible format?

Yes

Reviewer Expertise:

Neuroimaging, spinal cord injury.

I confirm that I have read this submission and believe that I have an appropriate level of expertise to confirm that it is of an acceptable scientific standard.

## References

[1] : Prognostic value of tissue bridges in cervical spinal cord injury: a longitudinal, multicentre, retrospective cohort study. *Lancet Neurol* .2024;23(8) : 10.1016/S1474-4422(24)00173-X 816-825 10.1016/S1474-4422(24)00173-X 38945142 PMC12128092

[2] : Spinal cord pathology revealed by MRI in traumatic spinal cord injury. *Curr Opin Neurol* .2021;34(6) : 10.1097/WCO.0000000000000998 789-795 10.1097/WCO.0000000000000998 34619692

# Reviewer report: Reviewer response for version 2

**DOI:** 10.5256/f1000research.168113.r294013

**Published:** 2024-07-08

**Authors:** Fabiano Reis

**Affiliations:** 1 University of Campinas (UNICAMP), Campinas, Brazil

There is a minor  mistake in the term Bechet's.

 The correct is *Behçet*

Is the study design appropriate for the research question?

Yes

Is the rationale for, and objectives of, the study clearly described?

Yes

Are sufficient details of the methods provided to allow replication by others?

Partly

Are the datasets clearly presented in a useable and accessible format?

Partly

Reviewer Expertise:

Neuroradiology

I confirm that I have read this submission and believe that I have an appropriate level of expertise to confirm that it is of an acceptable scientific standard.

# Response

**Published:** 2024-07-19

**Authors:** Rishabh Dhabalia

**Affiliations:** Datte Meghe Institute of Higher Education & Research, India

Thank you sir for your expert review. We'll rectify the mistake.

# Reviewer report: Reviewer response for version 1

**DOI:** 10.5256/f1000research.157864.r284747

**Published:** 2024-06-02

**Authors:** Fabiano Reis

**Affiliations:** 1 University of Campinas (UNICAMP), Campinas, Brazil

1. **An observational cross-sectional study of the role of magnetic resonance imaging in the evaluation of  the spinal cord in a tertiary care hospital in central India**

**The title is too long, I would suggest to remove some words. See above.**

**2.**Hyperintensities on T2-weighted images in the spinal cord (use this form in the background).

 3. In objectives, include:

- 6. To establish a diagnostic approach considering the topography and extension of the spinal cord abnormalities. 
- 4. include
- 3. **Diagnostic approach:** Establishing a systematic, algorithmic approach for assessing intramedullary T2-weighted hyperintensities, considering the topography and extension of the abnormalities.
- 5. USE THE WORD LESION This includes individuals with clinically diagnosed or suspected spinal cord lesion referred to the Department of Radiodiagnosis,
- 6. **MRI evaluation:** After enrollment, eligible patients will undergo an MRI evaluation of the spinal cord. The imaging will be performed per the standard protocols and procedures established by the Department of Radiodiagnosis. DETAIL THIS PROTOCOL
- 7. **Imaging data acquisition:** The core of this study relies on magnetic resonance imaging (MRI) to assess T2-weighted hyperintensities in the spinal cord. Imaging data will be collected using state-of-the-art MRI equipment following established protocols (DETAIL THE PROTOCOLS).
- 8.  AFTER CONDITIONS, INCLUDE THIS INFORMATION AND THIS RESPECTIVE RECENT REFERENCE including traumatic injuries, disc herniation, inflammatory, and demyelinating conditions.
  The main imaging patterns of demyelinating myelopathies (multiple sclerosis, neuromyelitis Optica spectrum disorder, acute disseminated encephalomyelitis, and myelin oligodendrocyte glycoprotein antibody-related disorder) and inflammatory myelopathies (systemic lupus erythematosus-myelitis, sarcoidosis-myelitis, Sjogren-myelitis, and Bechet's-myelitis) have MRI features that can aid to obtain the right diagnosis.
  Tamanini JVG, Sabino JV, Cordeiro RA, Mizubuti V, Villarinho LL, Duarte JÁ, Pereira FV, Appenzeller S, Damasceno A, Reis F. The Role of MRI in Differentiating Demyelinating and Inflammatory (not Infectious) Myelopathies. Semin Ultrasound CT MR. 2023 Oct;44(5):469-488.

Is the study design appropriate for the research question?

Yes

Is the rationale for, and objectives of, the study clearly described?

Yes

Are sufficient details of the methods provided to allow replication by others?

Partly

Are the datasets clearly presented in a useable and accessible format?

Partly

Reviewer Expertise:

Neuroradiology

I confirm that I have read this submission and believe that I have an appropriate level of expertise to confirm that it is of an acceptable scientific standard, however I have significant reservations, as outlined above.

## References

[1] : The Role of MRI in Differentiating Demyelinating and Inflammatory (not Infectious) Myelopathies. *Semin Ultrasound CT MR* .2023;44(5) : 10.1053/j.sult.2023.03.017 469-488 10.1053/j.sult.2023.03.017 37555683

# Response

**Published:** 2024-06-18

**Authors:** Rishabh Dhabalia

**Affiliations:** Datte Meghe Institute of Higher Education & Research, India

Thank you sir for your expert review. I have made the changes as per your advice.
